# Citrinin-Induced Hepatotoxicity in Mice Is Regulated by the Ca^2+^/Endoplasmic Reticulum Stress Signaling Pathway

**DOI:** 10.3390/toxins14040259

**Published:** 2022-04-06

**Authors:** Dongyi Wu, Chenglin Yang, Mengran Yang, You Wu, Yan Mao, Xinyan Zhou, Ji Wang, Zhihang Yuan, Jing Wu

**Affiliations:** 1 Colleges of Veterinary Medicine, Hunan Agricultural University, Changsha 410128, China; wdy1043530525@stu.hunau.edu.cn (D.W.); chenglin4698@163.com (C.Y.); ymr980218@stu.hunau.edu.cn (M.Y.); youyouwa0116@stu.hunau.edu.cn (Y.W.); y15757396115@163.com (Y.M.); zxy1085082373@163.com (X.Z.); wangjics@163.com (J.W.); 2 Hunan Engineering Research Center of Livestock and Poultry Health Care, Changsha 410128, China

**Keywords:** citrinin, endoplasmic reticulum stress, oxidative stress, apoptosis, hepatotoxicity, 4-phenylbutyric acid

## Abstract

Citrinin (CTN) is a mycotoxin found in crops and agricultural products and poses a serious threat to human and animal health. The aim of this study is to investigate the hepatotoxicity of CTN in mice and analyze its mechanisms from Ca^2+^-dependent endoplasmic reticulum (ER) stress perspective. We showed that CTN induced histopathological damage, caused ultrastructural changes in liver cells, and induced abnormal values of biochemical laboratory tests of some liver functions in mice. Treatment with CTN could induce nitric oxide (NO), malondialdehyde (MDA), and reactive oxygen species (ROS) accumulation in mice, accompanied with losses of activities of superoxide dismutase (SOD) and catalase (CAT), levels of glutathione (GSH), and capacities of total antioxidant (T-AOC), resulting in oxidative stress in mice. Furthermore, CTN treatment significantly increased Ca^2+^ accumulation, upregulated protein expressions of ER stress-mediated apoptosis signal protein (glucose regulated protein 78 (GRP78/BIP), C/EBP-homologous protein (CHOP), Caspase-12, and Caspase-3), and induced hepatocyte apoptosis. These adverse effects were counteracted by 4-phenylbutyric acid (4-PBA), an ER stress inhibitor. In summary, our results showed a possible underlying molecular mechanism for CTN that induced hepatocyte apoptosis in mice by the regulation of the Ca^2+^/ER stress signaling pathway.

## 1. Introduction

Mycotoxins are toxic secondary metabolites produced by molds, contaminating more than 25% of the world’s agricultural products (e.g., grains, fruits, and nuts) and causing serious threat to human and animal health. Thus, mycotoxins draws the wide attention of the international society as a global problem [1,2].

Citrinin (CTN) is a mycotoxin produced by Penicillium and Aspergillus in nature and causes a variety of toxic effects in animal and humans through oxidative stress [3,4]. The liver is the most important organ to metabolize toxins, and CTN causes hepatocytes damage by inducing ROS-mediated DNA damage and caspase-dependent endogenous apoptosis [5]. CTN not only exerts hepatotoxicity but also induces nephrotoxicity and reproductive toxicity [4,6,7]. Research indicates that CTN can affect mouse oocytes maturation via oxidative stress [5]. In addition, several studies have reported synergistic toxic effects of CTN with ochratoxin and deoxynivalenol [8].

ER is essential for protein synthesis, post-translational folding and modification, calcium homeostasis, and lipid metabolism. Under physiological condition, three endoplasmic reticulum kinases, inositol-requiring kinase 1 (IRE1), protein kinase R-like endoplasmic reticulum kinase (PERK), and activating transcription factor 6 (ATF6), are combined by a chaperone-binding immunoglobulin protein (BiP, also known as GRP78), thus being maintained in an inactive state. Both endogenous or exogenous stimuli, such as Ca^2+^ disorder, oxidative stress, hypoxia, and mycotoxin exposure, can activate ER stress. Upon ER stress, GRP78 is separated from the sensors, and UPR is initiated by activating IRE1α, PERK, and ATF6. Continued UPR activation will, therefore, lead to CHOP and apoptosis induction [9,10,11]. ER stress also plays an important role in liver injury induced by other mycotoxins, such as 3-Ac-DON exposure impairing the function of liver, as shown by oxidative damage, cell death, and the infiltration of immune cell, in which ER stress played an important role [11]. Fumonisin B1 induces ER stress-mediated hepatocyte toxicity by the IRE1α axis of URP, and oxidative stress and ER stress augmented each other through a positive feedback mechanism in the process [12]. Moreover, 4-PBA, an ER stress inhibitor, can significantly abolish 3-Ac-DON induced ER stress, oxidative damage, cell death, the infiltration of immune cells, and increased mRNA levels of inflammatory cytokines, indicating a critical role of UPR signaling for the cellular damage of the liver in response to mycotoxin exposure [11].

In this study, we used mice as an animal model to explore the toxic effects of CTN on liver and focused on how Ca^2+^/ER stress contributes to CTN-induced hepatocyte apoptosis.

## 2. Results

### 2.1. The Effects of CTN on Liver Injury

**Figure 1:**
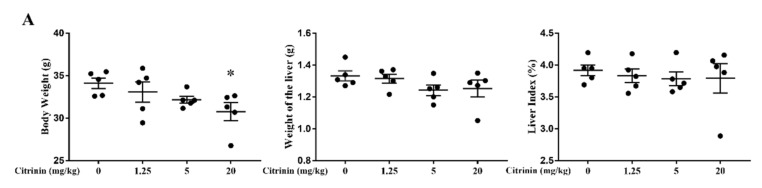

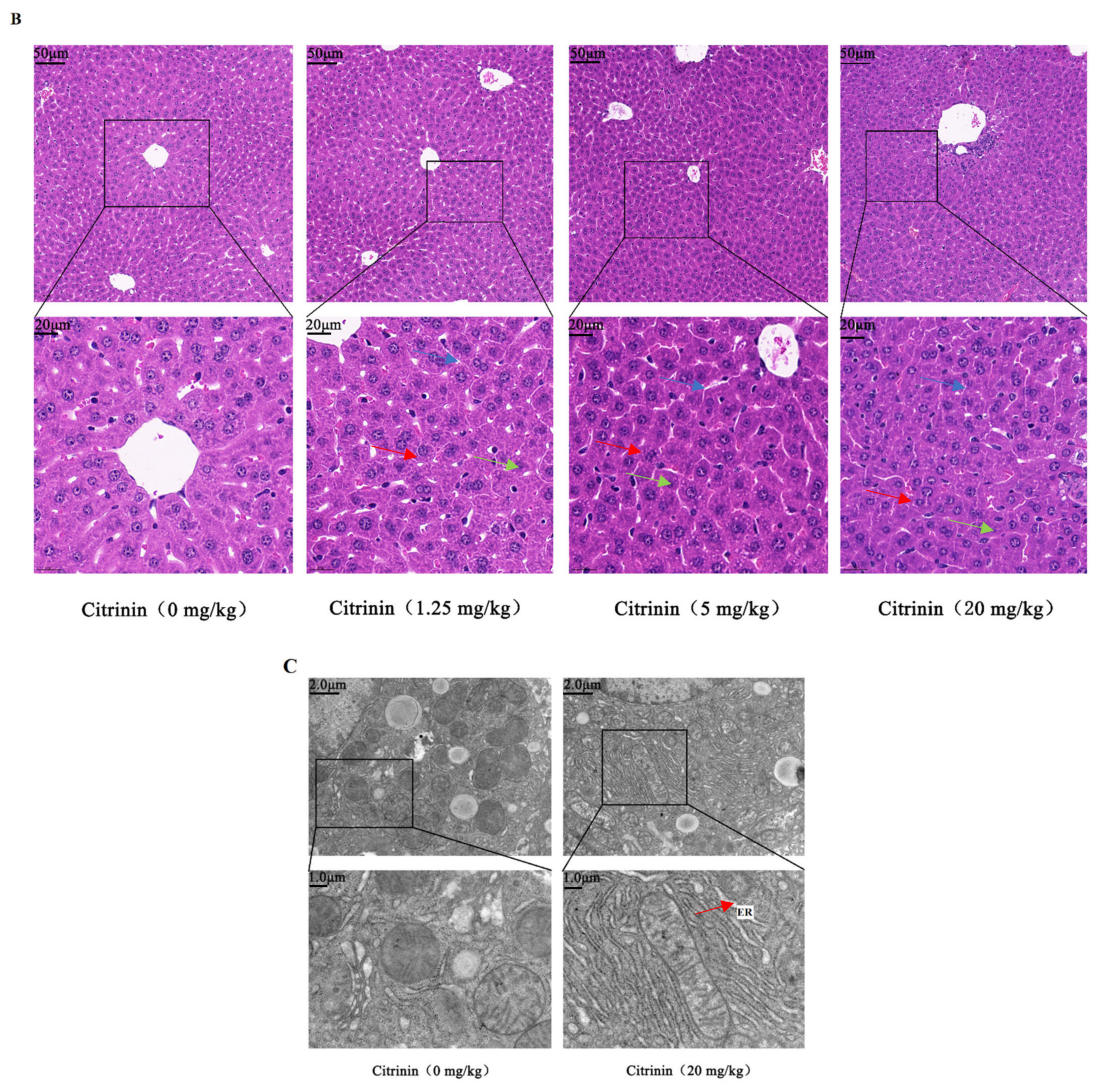
Effects of CTN on liver injury in mice. (**A**) Body weight, liver weight, and liver coefficient of mice exposed to different doses of CTN. (**B**) Evaluation of liver histopathological damage (Blue arrows indicate hepatic cord disorder, red arrows indicate hepatocyte swelling, and green arrows indicate dissolution. Scale bar: 20 μm). (**C**) Ultramicrostructure observation of liver tissue (red arrows indicate expansion of endoplasmic reticulum. Scale bar: 2 μm and 1 μm). The above data are expressed as mean ± SEM. Compared with the control group, * *p* < 0.05.

To explore the effects of CTN on liver injury, we observed mouse body weight, liver weight, liver coefficients, and histopathological and ultrastructural damage. CTN decreased the body weight of mice in a dose-dependent manner, and CTN at 20 mg/kg decreased significantly compared with the control group (*p* < 0.05). Moreover, there was no significant difference in liver weight between the control group and CTN group (*p* > 0.05), but the liver weight showed a decreasing trend with the increase in CTN concentration. Furthermore, compared with the control, the overall trend of liver index change was not obvious after CTN exposure (*p* > 0.05). To further confirm the impact of CTN on liver injury, histopathological and ultrastructural examinations showed that CTN significantly increased hepatic cord disorder, central venous congestion, nucleolysis, and ER dilation (Figure 1B,C). Therefore, CTN induced hepatotoxicity.

### 2.2. The Effects of CTN on Biochemical of Liver Functions

**Figure 2:**
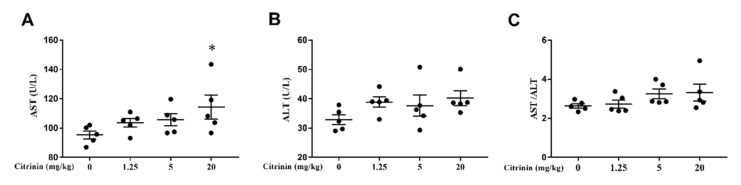
Effects of CTN on blood biochemical indexes in mice. The activities of AST (**A**), ALT (**B**), and AST/ALT ratio (**C**) in the blood of mice exposed to CTN were detected. The above data are expressed as mean ± SEM. Compared with the control group, * *p* < 0.05.

Clinically, serum alanine aminotransferase (ALT) and aspartate aminotransferase (AST) activities and AST/ALT ratios are important as liver function biochemical indicators. As the results are shown in Figure 2, the activities of AST and ALT and the AST/AST ratios increased with an increase in CTN concentrations, particularly the AST activity in the high dosage groups more than the control group (*p* < 0.05). It was suggested that CTN could affect liver functions in mice.

### 2.3. The Effects of CTN on Liver Oxidative Damage

**Figure 3:**
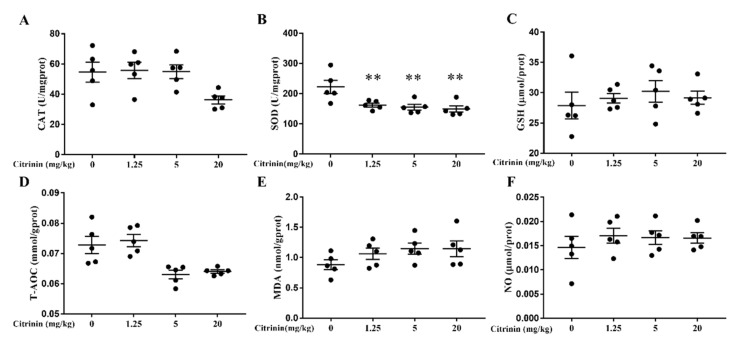

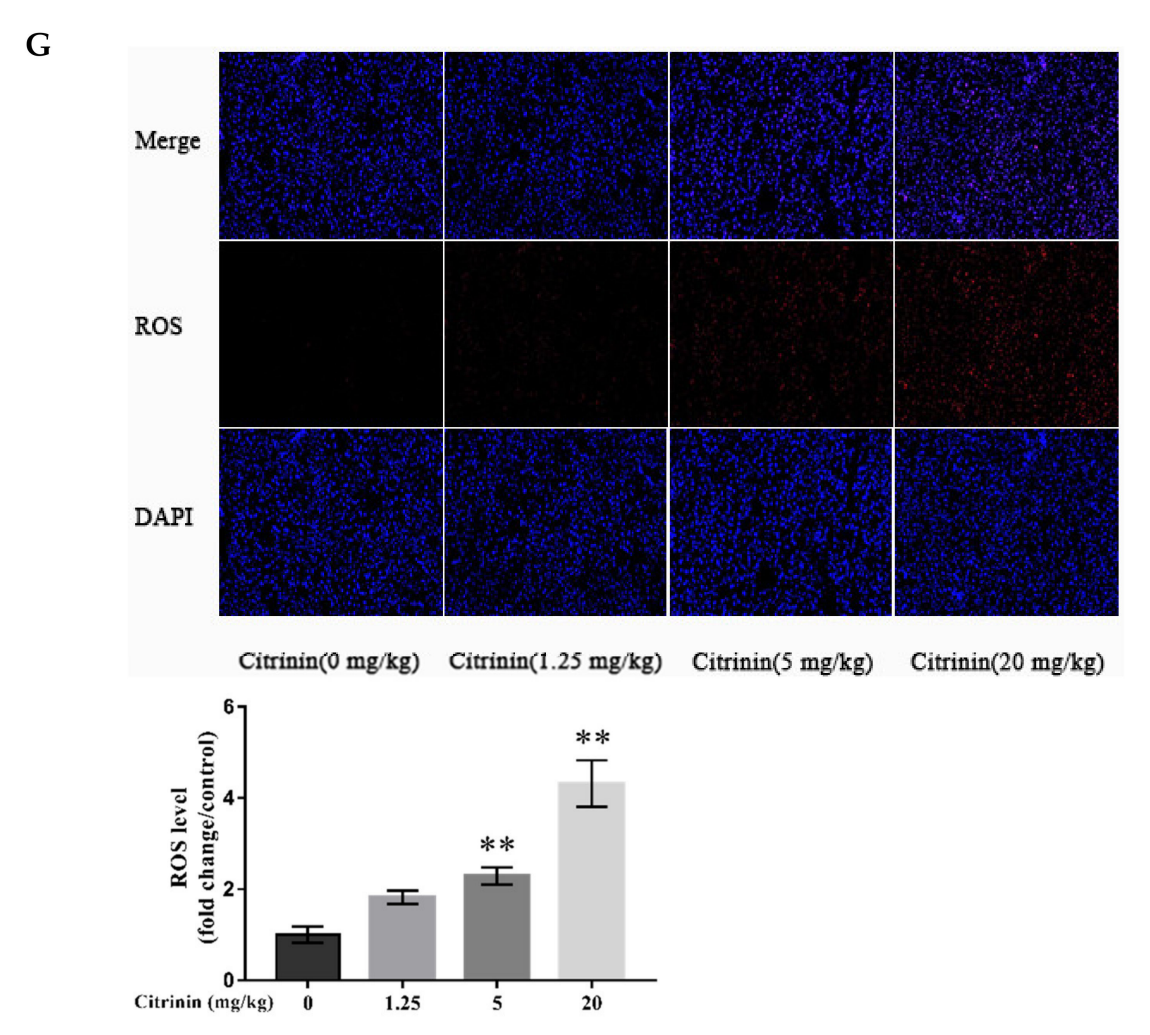
Effects of CTN on oxidative damage in the liver of mice. After all the liver tissues were processed, oxidation and antioxidant-related indicators show CAT (**A**), SOD (**B**), GSH (**C**), T-AOC (**D**), MDA (**E**), and NO (**F**). (**G**). ROS content in liver tissue was detected by fluorescence microscope (Figure scale bar: 100 μm). The above data are expressed as mean ± SEM. Compared with the control group, ** *p* < 0.01.

The production of ROS is mainly due to the imbalance between oxidation and anti-oxidation, which results in decreasing activities of antioxidant enzymes (CAT and SOD) with a decreasing antioxidant capacity as indicated by GSH levels and increases in oxidative damage as indicated by the increasing MDA, which is indicative of lipid peroxidation and NO, which is a radical compound. As shown in Figure 3A–C, CTN decreased the activities of CAT (*p* > 0.05) and SOD (*p* < 0.01) in a dose-dependent manner but showed no effect on GSH and NO contents (Figure 3C,F). It could reduce T-AOC levels (*p* > 0.05) and thereby increase MDA (*p* > 0.05) and ROS (*p* < 0.01) contents (Figure 3D,E,G) that ultimately caused oxidative damage in mice liver.

### 2.4. The Effects of CTN on Hepatocyte Apoptosis

**Figure 4:**
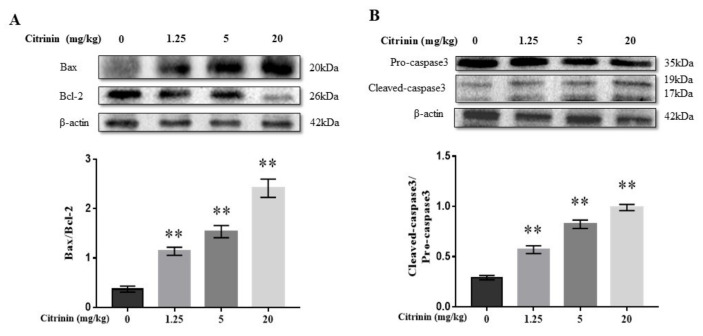
Effects of CTN on hepatocyte apoptosis in mice. The protein expression levels of Bax, Bcl-2 (**A**), and cleaved-caspase-3 and pro-caspase-3 (**B**) in liver tissue were detected by Western blot, and analyzed using image-J software. The above data are expressed as mean ± SEM. Compared with the control group, ** *p* < 0.01.

In many instances, apoptosis is involved in tissue damage, and Bax, Bcl-2 and caspase-3 have been confirmed to be important apoptosis-regulating proteins [13]. As shown in Figure 4, CTN significantly increased Bax/Bcl-2 and cleaved-caspase-3/pro-caspase-3 ratios in a dose-dependent manner (*p* < 0.01) (Figure 4), suggesting that CTN induced hepatocyte apoptosis.

### 2.5. The Effects of CTN on ER Stress

**Figure 5:**
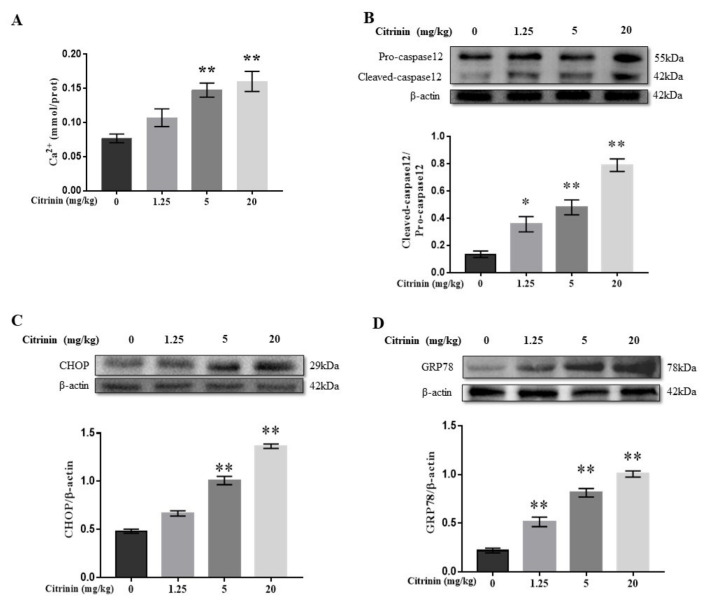
The effects of CTN on Ca^2+^ concentration and ER stress-associated proteins expression in the liver of mice. After the liver tissue was homogenized, the supernatant was taken to detect the change of Ca^2+^ content (**A**). The protein expression levels of caspase-12 (**B**), CHOP (**C**) and GRP78 (**D**) in liver tissue were detected by Western blot and analyzed using image-J software. The above data are expressed as mean ± SEM. Compared with the control group, ** *p* < 0.01, * *p* < 0.05.

To explore the role of ERs in hepatocyte apoptosis-induced by CTN, Ca^2+^ and ER stress-associated proteins (caspase-12, CHOP, and GRP78) in liver tissue were examined. Compared with the control group, Ca^2+^ concentration was significantly increased in the medium-dose and high-dose groups (*p* < 0.01), and there was no significant difference in low dose (*p* < 0.05), suggesting calcium homeostasis disorder (Figure 5A). The expressions of GRP78 and CHOP and ration of cleaved caspase-12/pro-caspase-12 were significantly upregulated in a dose-dependent manner (Figure 5B–D). Thus, CTN induced hepatocyte apoptosis, which might be related to ER stress.

### 2.6. The Effects of 4-PBA on ER Stress Mediated Hepatocyte Apoptosis-Induced by CTN

**Figure 6:**
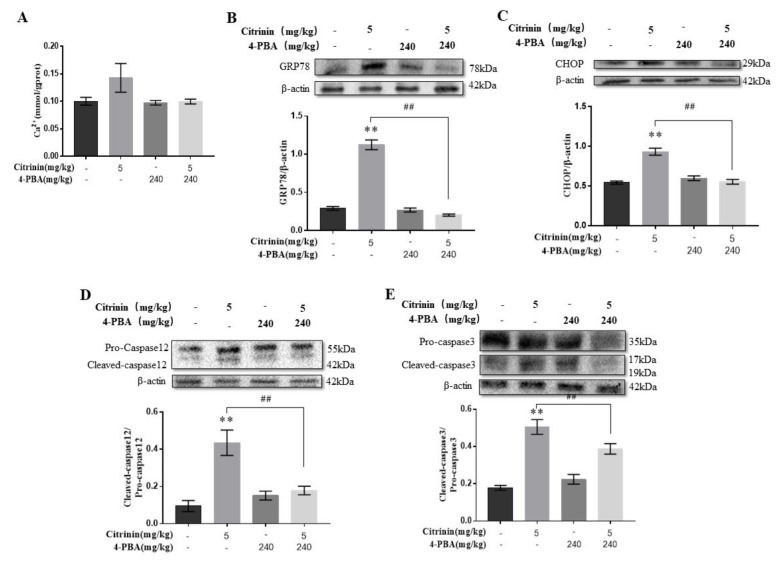
The effects of 4-PBA on ER stress-mediated hepatocyte apoptosis-induced by CTN in mice liver. After the liver tissue was homogenized, the supernatant was taken to detect the change of calcium ion content (**A**). The protein expressions of GRP78 (**B**), CHOP (**C**), caspase-12 (**D**), and caspase-3 (**E**) in liver tissue were detected by Western blot and analyzed using image-J soft. The above data are expressed as mean ± SEM. Compared with the control group, ** *p* < 0.01. Compared with the Citrinin (5 mg/kg) group, ## *p* < 0.01.

To further define the role of ER stress in CTN-induced apoptosis, mice were treated with ER stress inhibitor 4-PBA (240 mg/kg) in the CTN (5 mg/kg) treatment. The results showed that 4-PBA reversed the CTN effects on Ca^2+^ concentration, CHOP and GRP78 protein expression, and caspase-3/12 activation in mice liver (Figure 6), suggesting that ER stress played an important regulatory role in hepatocyte apoptosis induced by CTN.

### 2.7. The Effects of 4-PBA on CTN-Induced Oxidative Stress in Liver

**Figure 7:**
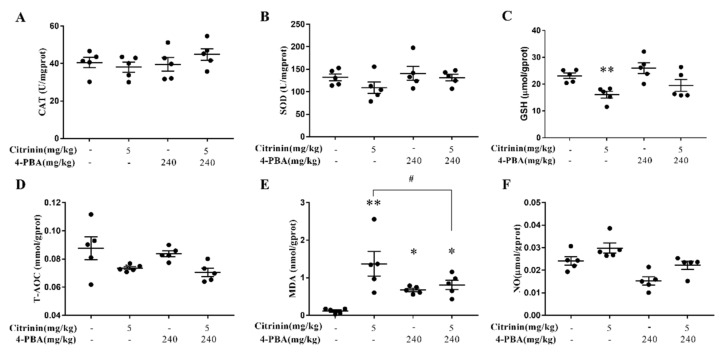

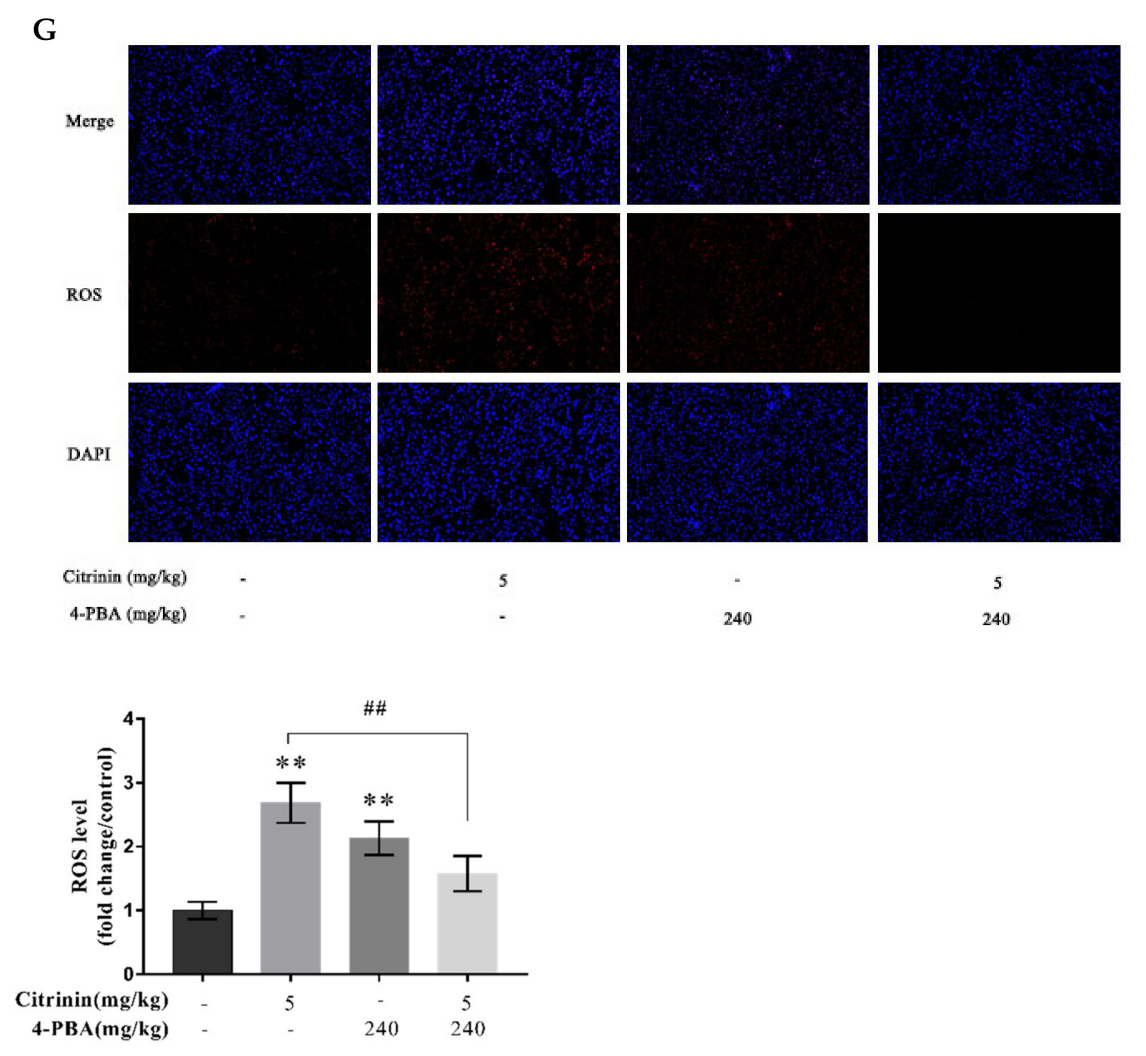
The effects of 4-PBA on CTN-induced oxidative stress in liver. After the liver tissue was homogenized, the supernatant was taken to detect CAT (**A**), SOD (**B**), GSH (**C**), T-AOC (**D**), MDA (**E**), and NO (**F**); (**G**) ROS in liver tissue was detected by fluorescence microscope (Figure scale bar: 100 μm). The above data are expressed as mean ± SEM. Compared with the control group, ** *p* < 0.01, * *p* < 0.05. Compared with the Citrinin (5 mg/kg) group, # *p* < 0.05, ## *p* < 0.01.

In our study, co-treatment with 4-PBA reversed the CTN-induced inhibition of the CAT (Figure 7A) and SOD (Figure 7B) activity. Moreover, 4-PBA co-treatment with CTN increased GSH (Figure 7C) and T-AOC (Figure 7D) levels with concomitant decreases in MDA (Figure 7E) and NO (Figure 7F).

### 2.8. The Effects of 4-PBA Alleviates CTN-Induced Liver Injury

**Figure 8:**
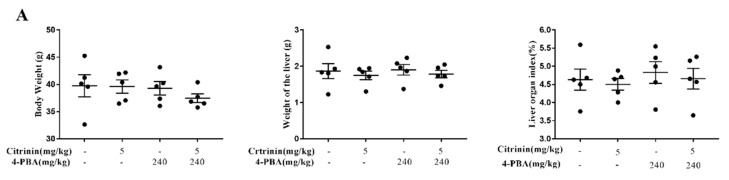

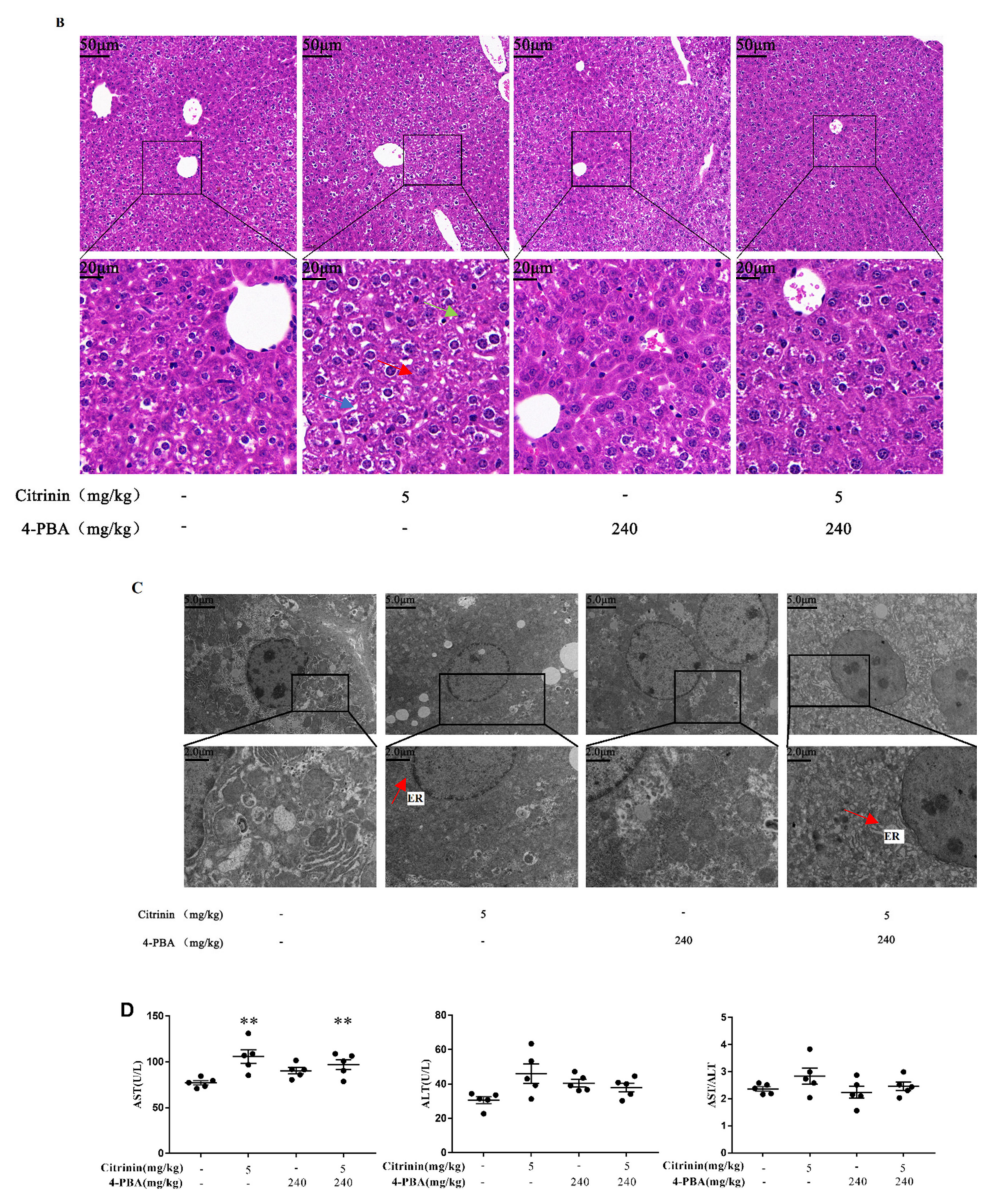
The effects of 4-PBA alleviates CTN-induced liver injury. (**A**) Body weight, liver weight, and liver coefficient of mice. (**B**) Histomorphological observation of liver (blue arrows indicate hepatic cord disorder, red arrows indicate hepatocyte swelling, and green arrows indicate nuclear lysis. Scale bar: 50 and 20 μm). (**C**) Ultramicrostructure observation of liver tissue (red arrows indicate expansion of endoplasmic reticulum. Scale bar: 2 μm and 1 μm). (**D**). The activities of AST, ALT, and AST/ALT ratio were detected. The above data are expressed as mean ± SEM. Compared with the control group, ** *p* < 0.01.

In our study, co-treatment with 4-PBA alleviated CTN-induced hepatic cord disorder, hepatocyte enlargement, nucleolysis, and ER expansion (Figure 8B,C) and then reversed AST and ALT activities and the AST/ALT ratio (Figure 8D), but it showed no effects on body weight, liver weight, and liver coefficients (Figure 8A), suggesting that the inhibition of ER stress could alleviate CTN-induced liver injury.

## 3. Discussion

Mycotoxins are metabolized and detoxicated in the liver (one of the largest digestive organs in the body) after being digested and absorbed into the body [14,15], so the liver is the main target organ of mycotoxins.

In clinical practice, the liver index and AST/ALT ratio can be parameters for the assessment liver function [16]. In our study, with increasing CTN dosages, there was obviously hepatocyte swelling (Figure 1B), and the AST/ALT ratio increased (Figure 2), suggesting liver injury (Figure 2). Oxidative stress, a disturbance in the balance between the production of reactive oxygen species (free radicals) and antioxidant defenses, is a key molecular process in cell damage and also a major factor of toxic effects induced by mycotoxins [17,18]. SOD, CAT, and GSH are the main components of the body’s antioxidant system, reflecting the body’s ability to remove free radicals [19]. Under pathological conditions, ROSs are produced in concentrations that cannot be controlled by the usual protective antioxidant mechanisms employed by the cells, resulting in a decrease in total antioxidant capacity. NO is one of the nitrogenous radical, the possible consequences of free radical damage are provided with special emphasis on lipid peroxidation, and the main product of lipid peroxidation is MDA. Previous studies showed that deoxynivalenol induced oxidative stress in mouse liver by increasing MDA levels in tissues [20]. Ochratoxin A can aggravated renal oxidative stress by reducing CAT, SOD, and glutathione peroxidase (GSH-Px) activity [21]. CTN induced the increase in ROS in rat hepatocytes, stimulates the production of superoxide anions in the respiratory chain and inducing oxidative damage in the rat liver [22]. In our study, similar results were observed, and CTN significantly reduced CAT and SOD activities (Figure 3A,B) but showed no effect on GSH and NO contents (Figure 3C,F), indicating that T-AOC decreased (Figure 3D) and that there was an oxidation and antioxidant system imbalance. Due to the reduced activity of antioxidant scavenging enzymes, ROS increased significantly (Figure 3G) and caused lipid peroxidation, and the MDA concentration increased (Figure 3E), resulting in oxidative stress in hepatocytes after mice exposure to CTN.

Numerous studies have shown that oxidative stress could also trigger apoptosis [23,24,25]. From the view of histomorphology, apoptosis mainly shows nucleolysis or karyorrhexis, as shown as Figure 1. The classical systems inducing apoptosis are divided into endogenous (ER stress and mitochondrial) and exogenous (death receptor) pathways. The endogenous pathway is mainly regulated by BCL-2 family proteins (such as Bcl-2, Bax, etc.), and the exogenous pathway is mainly regulated by cysteine protease (caspase) [13,26]. Anti-apoptotic proteins such as Bcl-2 inhibit apoptosis by blocking the expression of pro-apoptotic protein (Bax) [27]. Bcl-2 can also act as a direct substrate of Caspase-3, Caspase-3 acts as a physiological enzymatic protease of Bcl-2, and the two interact and restrict each other [28]. When Bax/Bcl-2 ratio increased, downstream Caspase-3 was activated and apoptosis was induced [29]. Previous research shows that zearalenone upregulates the expression of Bax, Bcl-2, and apoptotic executive protein cleaved caspase-3 by increasing MDA content and GSH and CAT activities, resulting in the apoptosis of mouse ovarian cells [30]. Thus, zearalenone can be seen to play an important role of oxidative stress-mediated apoptosis in mycotoxin induced toxic effects. Additionally, T-2 toxin induced apoptosis in human hepatocytes by regulating the expression of Bax and Bcl-2 and upregulating the expression of cleaved-caspase-3 [31], aflatoxin B1, and fumonisin B1-induced hepatocyte apoptosis in mice, where Bax and cleaved-caspase-3 expressions were upregulated and Bcl-2 expression was downregulated [32,33]. In our study, CTN significantly increased Bax/Bcl-2 and cleaved-caspase-3/pro-caspase-3 ratios in a dose-dependent manner, indicating that CTN also induced hepatocyte apoptosis.

Meanwhile, we also found that CTN induced ER dilation in the liver of mice (Figure 1C). The ER is usually relatively stable, but oxidative stress, calcium disturbance, and misfolded proteins accumulate, and imbalanced ER homeostasis have been reported to activate ER stress, initiating folding protein responses and caspase-12 dependent apoptosis [33,34,35]. It is worthy to note that Ca^2+^ disorder breaks the balance between Bcl-2 and Bax, resulting in ER-associated degradation (ERAD); thus, maintaining Ca^2+^/ER homeostasis is essential for most ER functions [35,36,37,38,39,40]. In our study, CTN significantly increased Ca^2+^ levels in the liver (*p* < 0.01), suggesting a disturbance of homeostasis in calcium signaling systems. Thus, we hypothesized CTN-induced liver damage in mice was also related to Ca^2+^/ER stress signaling. GRP78 (BIP), CHOP, and Caspase-12 are regarded as markers of ER stress. BIP is a particularly critical Ca^2+^-binding protein that is mainly involved in Ca^2+^ transport and the regulation of folding protein reactions [41,42,43]. Interaction between BIP and ER stress sensor could regulate Ca^2+^ from exuding from endoplasmic reticulum, and the interaction plays a key role in maintaining ER homeostasis [39]. CHOP is activated when BIP binds to misfolded proteins, can regulate the caspase-12, BCL-2 family proteins (such as Bcl-2) and caspase-3 [44,45,46,47]. Caspase-12, one of the cysteine proteases, can activate Caspase-3 through ER stress, thus inducing apoptosis activated by ER stress and promotes apoptosis [48,49]. Several studies reported that mycotoxins induced apoptosis by activating ER stress [50,51]. For example, zearalenone induced mouse testicular interstitial cell damage, where GRP78, CHOP, and Caspase-12 expressions were significantly upregulated [52]. Similar data were also observed in the process of patulin induced apoptosis of intestinal and renal cells [53]. In our study, CTN significantly increased Ca^2+^ level (*p* < 0.01), which suggests an imbalanced calcium homeostasis in the liver concomitant with significantly increased Caspase-12, CHOP, and GRP78 expressions in mice liver (*p* < 0.01) (Figure 5). It was concluded that Ca^2+^/ER stress was involved in CTN-induced hepatocyte apoptosis.

**Figure 9:**
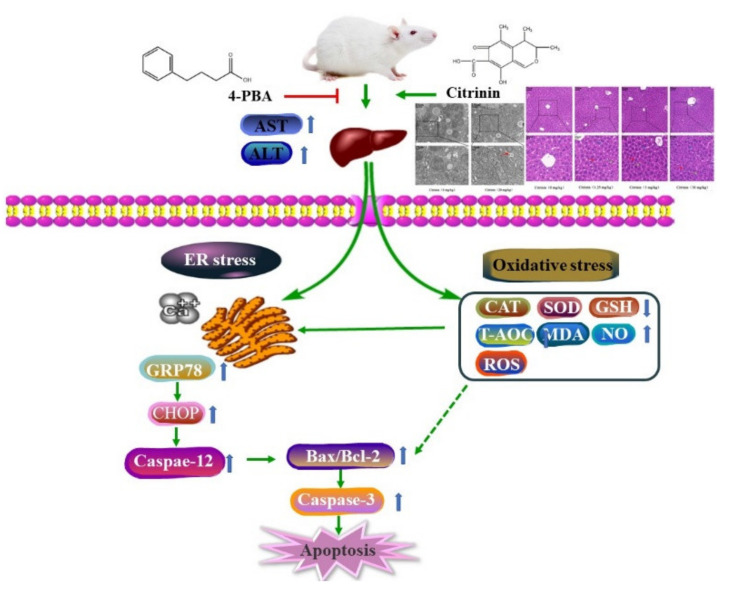
Citrinin-induced hepatotoxicity in mice is regulated by the Ca^2+^/endoplasmic reticulum stress signaling pathway.

To further clarify Ca^2+^/ER stress in CTN-induced liver injury, mice were treated with ER stress inhibitor 4-PBA (240 mg/kg) during CTN (5 mg/kg) treatment. 4-PBA is an effective ER stress inhibitor and has been used in clinical trials to treat diseases where misfolded proteins accumulate in cells [54]. In our study, compared with the CTN group, co-treatment with 4-PBA significantly reduced Ca^2+^ levels to restore calcium homeostasis, downregulated ER stress markers (GRP78, CHOP, and Caspase-12), and decreased Bax/Bcl-2 and cleaved-caspase-3/pro-caspase-3 ratios (*p* < 0.01) (Figure 9). This is consistent with the fact that 4-PBA can inhibit nephrotoxicity caused by the T-2 toxin via ER stress [44] and hepatotoxicity mediated by 3-Ac-DON [11]. Additionally, CTN induced liver tissue damage (Figure 9), and oxidative stresses (Figure 7) were alleviated after 4-PBA co-treatment, which is consistent with 4-PBA reversed 3-acetyldeoxynivalenol residue levels, decreased hepatic CAT and SOD activities, and increased MDA levels, thereby alleviating liver injury in mice [13]. Eventually, we believe that the Ca^2+^/ER stress signaling pathway plays a key regulatory role in CTN-induced mice liver injury.

## 4. Conclusions

In conclusion, our results suggest that CTN can induce oxidative stress and Ca^2+^ disorder, resulting in ER stress-mediated apoptosis and eventually causing liver damage. These adverse effects were counteracted by 4-phenylbutyric acid (4-PBA), an ER stress inhibitor. It proves that the Ca^2+^/ER stress signaling pathway plays a key regulatory role in CTN-induced mice liver injury, as shown in Figure 9. The findings compensate the mechanisms of CTN-induced liver injury and provide a new therapeutic target to treat the damage by CTN.

## 5. Materials and Methods

### 5.1. Drugs and Reagents

CTN (MSS1009) was provided by Pribolab (Qingdao, Shandong, China). 4-PBA was purchased from Sigma Commercial (St Louis, MO, USA). Glutathione (GSH) (A006-2-1), malondialdehyde (MDA) (A003-1-2), superoxide dismutase (SOD) (A001-3-2), catalase (CAT) (A007-1-1), nitric oxide (NO) (A013-2-1), calcium ion (C004-2-1), and bicinchoninic acid (BCA) assay kits (A045-4-2) were purchased from Nanjing Jian Cheng Bioengineering Institute (Nanjing, Jiangsu, China). Hematoxylin and eosin (H&E) staining solution (G1005) was purchased from Wuhan Servicebio (Wuhan, Hubei, China). Hypersensitive ECL chemiluminescence Kit (P10100) was purchased from NCM Biotech (Suzhou, Jiangsu, China). Bax (380709) and Bcl-2 (381702) antibodies were provided by Chengdu Positive Energy biology (Chengdu, China). The Caspase-12 (35965S) antibody was provided by Cell Signaling Technology Inc. (Danvers, MA, USA); CHOP (15204-1-AP), GRP78 (11587-1-AP), Caspase-3 (19677-1-AP), and Beta Actin (20536-1-AP) antibodies were provided by Proteintech (Wuhan, China). Therm (Fementas) offered the protein marker (26616) (Burlington, VT, USA).

### 5.2. Animals and Treatments

Twenty SPF-grade Kunming (KM) male mice (6 weeks old and weighing 25 ± 2 g) were purchased from Hunan SJA Laboratory Animal Co., Ltd (license No.: SCXK (Xiang) 2019-0004) and animals were housed in the mouse room at the College of Animal Medicine, Hunan Agricultural University with a 12 h circadian light cycle, with free access to food and water, temperature = 23 ± 3 °C, and humidity = 50–70%. Mice were routinely fed for 1 week (Changsha, Hunan, China) and then randomly divided into four groups (*n* = 20): (1) blank group (*n* = 5), (2) low (*n* = 5), (3) medium (*n* = 5), and (4) high CTN groups (*n* = 5) (1.25 mg/kg, 5 mg/kg, and 20 mg/kg, respectively). CTN is mainly dissolved in anhydrous ethanol and diluted with double steaming water in accordance with the corresponding proportion (the final anhydrous ethanol concentration is 2%). The blank group was given 2% ethanol with gavage administration. The low, medium, and high CTN groups were perfused with different doses of CTN each day for 14 days.

The twenty SPF-grade Kunming (KM) male mice (6 weeks old and weighing 26 ± 2 g) were divided into four groups (*n* = 20): (1) blank group (*n* = 5), (2) 5 mg/kg CTN group (*n* = 5), (3) 4-PBA group (*n* = 5), and (4) 4-PBA + CTN group (*n* = 5). After raising the mice conventionally for one week, 4-PBA was injected intraperitoneally, and CTN was administrated orally once a day for 2 weeks. The blank group was given 2% ethanol. The CTN group was provided with 5 mg/kg CTN. The 4-PBA group was administrated with 2% ethanol and injected with 240 mg/kg 4-PBA. The 4-PBA + CTN group was administrated with 5 mg/kg CTN and injected with 240 mg/kg 4-PBA. After fasting for 12 h on day 14 (water was allowed), Orbital blood sampling was performed in mice after the intraperitoneal injection of anesthetics and mice humanely euthanized by cervical vertebrae dislocation.

This experiment was carried out in accordance with the Guidelines for animal protection and use in China and approved by the Animal Protection Committee.

### 5.3. Measuring Liver Indices

Mouse growth was observed throughout the feeding period. Mouse and liver weights were recorded and liver indices calculated using the following formula.

Liver Index (%) = Liver Weight (g)/Body weight (g)*100%(1)

### 5.4. Liver Function Biochemical Indices

The blood samples were placed at 25 °C for 2 h followed by staying overnight at 4 °C and centrifuged for serum isolation. Liver-related indices in mouse serum were measured and analyzed using an automatic biochemical analyzer: Alanine aminotransferase (ALT), aspartate aminotransferase (AST), the ratio of the aspartate aminotransferase, and Alanine aminotransferase (AST/ALT).

### 5.5. Oxidation-Antioxidant Index Detection

Liver tissues measuring 0.1 g from each group were diluted 10 times by normal saline for tissue homogenization. The tissues were centrifugated followed by collecting the supernatant for further analysis. The levels of GSH, MDA, CAT, SOD, and NO in liver tissues were detected and calculated using kit instructions from Nanjing Jian Cheng Institute of Biological Engineering (Nanjing, China).

### 5.6. Detection of Ca^2+^ Content

The liver tissues were homogenized with lysis solution (RIPA: PMSF = 1:9) for complete lysis. After centrifugation, the content of Ca^2+^ in the superior solution was measured according to the protocol of the detection kit from Nanjing Jian Cheng Institute of Biological Engineering (Nanjing, China).

### 5.7. ROS Release Determination

The fresh liver tissues were subjected to fast freeze with liquid nitrogen. After embedding with an optimal cutting temperature (OCT), the tissues were sliced followed by incubating with 20, 70-dichlorofluorescin diacetate (DCFH-DA) in the dark for 30 min. PBS was added for washing, then DAPI was used to stained nuclear. Fluorescence was observed by fluorescence microscope (Tokyo, Japan) after sealing the slices.

### 5.8. Structural Observations of Liver Histology

Excised livers were placed in a universal tissue fixative, which was replaced after 24 h. After 72 h, tissues were processed by conventional histological techniques, sectioned, stained in hematoxylin and eosin (H&E), sealed with neutral gum, and observed microscopically, photographed, and recorded (Nikon Eclipse Ci, Tokyo, Japan).

### 5.9. Transmission Electron Microscope (TEM) Ultra-Structural Observations

Fresh tissues were immersed in 2.5% glutaraldehyde fixative for 3 h. After dehydration, samples were placed in 100% dehydrating agent and an equal amount of embedding agent and double stained overnight in uranyl acetate and lead citrate at room temperature. Morphological changes in liver cells were observed and photographed using TEM (H-7500, Hitachi, Tokyo, Japan).

### 5.10. Western Blotting

Proteins were extracted from 0.1 g liver tissue, separated using sodium dodecyl sulfate-polyacrylamide, and transferred (300 mA for 60 min) to methanol-activated polyvinylidene fluoride membranes (Millipore, Bedford, MA, USA). The respective antibodies were applied overnight and washed three times TBST (Wuhan, Hubei, China) on the next day. A secondary antibody was applied for 1 h and washed three times in TBST for 3 times. ECL was used for protein imaging and development (Alpha Innotech, San Leandro, CA, USA).

### 5.11. Data Analysis and Processing

One-way ANOVA was performed in SPSS 27.0 software (Release 27.0; SPSS, Inc., Chicago, IL, USA) to compare significant differences between groups. Graph pad Prism 7.0 software (GraphPad, Inc., La Jolla, San Diego, CA, USA) was used to graph data. Where appropriate, * and ** represented significant differences (*p* < 0.05) and highly significant differences (*p* < 0.01) reported to the bank group. The mice pretreated with 4-PBA at 5 mg/kg CTN were compared with the CTN group, and # and ##, respectively, represented significant difference (*p* < 0.05) and extremely significant difference (*p* < 0.01).

## Data Availability

Not applicable.

## References

[1] Turner P.C., Flannery B., Isitt C., Ali M., Pestka J. (2012). The role of biomarkers in evaluating human health concerns from fungal contaminants in food. Nutr. Res. Rev..

[2] Schmidt J., Cramer B., Turner P.C., Stoltzfus R.J., Humphrey J.H., Smith L.E., Humpf H.U. (2021). Determination of Urinary Mycotoxin Biomarkers Using a Sensitive Online Solid Phase Extraction-UHPLC-MS/MS Method. Toxins.

[3] He Y., Cox R.J. (2016). The molecular steps of citrinin biosynthesis in fungi. Chem. Sci..

[4] De Oliveira Filho J.W.G., Islam M.T., Ali E.S., Uddin S.J., Santos J.V.O., de Alencar M.V.O.B., Júnior A.L.G., Paz M.F.C.J., de Brito M.D.R.M., ESousa J.M.C. (2017). A comprehensive review on biological properties of citrinin. Food Chem. Toxicol..

[5] Gayathri L., Dhivya R., Dhanasekaran D., Periasamy V.S., Alshatwi A.A., Akbarsha M.A. (2015). Hepatotoxic effect of ochratoxin A and citrinin, alone and in combination, and protective effect of vitamin E: In vitro study in HepG2 cell. Food Chem. Toxicol..

[6] Ning Z.Q., Cui H., Xu Y., Huang Z.B., Tu Z., Li Y.P. (2017). Deleting the citrinin biosynthesis-related gene, ctnE, to greatly reduce citrinin production in Monascus aurantiacus Li AS3.4384. Int. J. Food Microbiol..

[7] Degen G.H., Ali N., Gundert-Remy U. (2018). Preliminary data on citrinin kinetics in humans and their use to estimate citrinin exposure based on biomarkers. Toxicol. Lett..

[8] Leonard A., Paton A.W., El-Quadi M., Paton J.C., Fazal F. (2014). Preconditioning with endoplasmic reticulum stress ameliorates endot- helial cell inflammation. PLoS ONE.

[9] Hwang J., Qi L. (2018). Quality Control in the Endoplasmic Reticulum: Crosstalk between ERAD and UPR pathways. Trends Biochem. Sci..

[10] Ghosh R., Colon-Negron K., Papa F.R. (2019). Endoplasmic reticulum stress, degeneration of pancreatic islet β-cells, and therapeutic modulation of the unfolded protein response in diabetes. Mol. Metab..

[11] Jia H., Liu N., Zhang Y., Wang C., Yang Y., Wu Z. (2021). 3-Acetyldeoxynivalenol induces cell death through endoplasmic reticulum stress in mouse liver. Environ. Pollut..

[12] Liu X., Zhang E., Yin S., Zhao C., Fan L., Hu H. (2020). Activation of the IRE1α Arm, but not the PERK Arm, of the Unfolded Protein Response Contributes to Fumonisin B1-Induced Hepatotoxicity. Toxins.

[13] Luo Y., Fu X., Ru R., Han B., Zhang F., Yuan L., Men H., Zhang S., Tian S., Dong B. (2020). CpG Oligodeoxynucleotides Induces Apoptosis of Human Bladder Cancer Cells via Caspase-3-Bax/Bcl-2-p53 Axis. Arch. Med. Res..

[14] Grant D.M. (1991). Detoxification pathways in the liver. J. Inherit. Metab. Dis..

[15] Reinke H., Asher G. (2016). Circadian Clock Control of Liver Metabolic Functions. Gastroenterology.

[16] Bennett J.W., Klich M. (2003). Mycotoxins. Clin. Microbiol. Rev..

[17] Islam M.T., Mishra S.K., Tripathi S., de Alencar M.V.O.B., ESousa J.M.C., Rolim H.M.L., de Medeiros M.D.G.F., Ferreira P.M.P., Rouf R., Uddin S.J. (2018). Mycotoxin-assisted mitochondrial dysfunction and cytotoxicity: Unexploited tools against proliferative disorders. IUBMB Life.

[18] Huang B., Chen Q., Wang L., Gao X., Zhu W., Mu P., Deng Y. (2020). Aflatoxin B1 Induces Neurotoxicity through Reactive Oxygen Species Generation, DNA Damage, Apoptosis, and S-Phase Cell Cycle Arrest. Int. J. Mol. Sci..

[19] Bahrehmand F., Vaisi-Raygani A., Kiani A., Rahimi Z., Tavilani H., Navabi S.J., Shakiba E., Hassanzadeh N., Pourmotabbed T. (2012). Matrix metalloproteinase-2 functional promoter polymorphism G1575A is associated with elevated circulatory MMP-2 levels and increased risk of cardiovascular disease in systemic lupus erythematosus patients. Lupus.

[20] Yu M., Peng Z., Liao Y., Wang L., Li D., Qin C., Hu J., Wang Z., Cai M., Cai Q. (2019). Deoxynivalenol-induced oxidative stress and Nrf2 translocation in maternal liver on gestation day 12.5 d and 18.5 d. Toxicon.

[21] Marin D.E., Pistol G.C., Gras M., Palade M., Taranu I. (2018). A comparison between the effects of ochratoxin A and aristolochic acid on the inflammation and oxidative stress in the liver and kidney of weanling piglets. Naunyn Schmiedebergs Arch. Pharmacol..

[22] Ribeiro S.M., Chagas G.M., Campello A.P., Klüppel M.L. (1997). Mechanism of citrinin-induced dysfunction of mitochondria. V. Effect on the homeostasis of the reactive oxygen species. Cell Biochem. Funct..

[23] Pei X., Jiang H., Liu X., Li L., Li C., Xiao X., Li D., Tang S. (2021). Targeting HMGB1 inhibits T-2 toxin-induced neurotoxicity via regulation of oxidative stress, neuroinflammation and neuronal apoptosis. Food Chem. Toxicol..

[24] Wang J., Jin Y., Wu S., Yu H., Zhao Y., Fang H., Shen J., Zhou C., Fu Y., Li R. (2019). Deoxynivalenol induces oxidative stress, inflammatory response and apoptosis in bovine mammary epithelial cells. J. Anim. Physiol. Anim. Nutr..

[25] Yang J., Guo W., Wang J., Yang X., Zhang Z., Zhao Z. (2020). T-2 Toxin-Induced Oxidative Stress Leads to Imbalance of Mitochondrial Fission and Fusion to Activate Cellular Apoptosis in the Human Liver 7702 Cell Line. Toxins.

[26] D’Arcy M.S. (2019). Cell death: A review of the major forms of apoptosis, necrosis and autophagy. Cell Biol. Int..

[27] Chen M., Zhou B., Zhong P., Rajamanickam V., Dai X., Karvannan K., Zhou H., Zhang X., Liang G. (2017). Increased Intracellular Reactive Oxygen Species Mediates the Anti-Cancer Effects of WZ35 via Activating Mitochondrial Apoptosis Pathway in Prostate Cancer Cells. Prostate.

[28] Kirsch D.G., Doseff A., Chau B.N., Lim D.S., de Souza-Pinto N.C., Hansford R., Kastan M.B., Lazebnik Y.A., Hardwick J.M. (1999). Caspase-3-dependent cleavage of Bcl-2 promotes release of cytochrome c. J. Biol. Chem..

[29] Muñoz-Pinedo C., Guío-Carrión A., Goldstein J.C., Fitzgerald P., Newmeyer D.D., Green D.R. (2006). Different mitochondrial intermembrane space proteins are released during apoptosis in a manner that is coordinately initiated but can vary in duration. Proc. Natl. Acad. Sci. USA.

[30] Wu J., Li J., Liu Y., Liao X., Wu D., Chen Y., Liang Z., Yuan Z., Li R., Yi J. (2021). Tannic acid repair of zearalenone-induced damage by regulating the death receptor and mitochondrial apoptosis signaling pathway in mice. Environ. Pollut..

[31] Wu J., Zhou Y., Yuan Z., Yi J., Chen J., Wang N., Tian Y. (2019). Autophagy and Apoptosis Interact to Modulate T-2 Toxin-Induced Toxicity in Liver Cells. Toxins.

[32] Li X., Lv Z., Chen J., Nepovimova E., Long M., Wu W., Kuca K. (2021). Bacillus amyloliquefaciens B10 can alleviate liver apoptosis and oxidative stress induced by aflatoxin B1. Food Chem. Toxicol..

[33] Bhandari N., Raghubir P., Sharma (2002). Modulation of selected cell signaling genes in mouse liver by fumonisin B1. Chem. Biol. Interact..

[34] West S.J., Kodakandla G., Wang Q., Tewari R., Zhu M.X., Boehning D., Akimzhanov A.M. (2022). S-acylation of Orai1 regulates store-operated Ca^2+^ entry. J. Cell Sci..

[35] Tadic V., Prell T., Lautenschlaeger J., Grosskreutz J. (2014). The ER mitochondria calcium cycle and ER stress response as therapeutic targets in amyotrophic lateral sclerosis. Front. Cell. Neurosci..

[36] Guerrero-Hernandez A., Verkhratsky A. (2014). Calcium signalling in diabetes. Cell Calcium.

[37] Collins H.E., Zhu-Mauldin X., Marchase R.B., Chatham J.C. (2013). STIM1/Orai1-mediated SOCE: Current perspectives and potential roles in cardiac function and pathology. Am. J. Physiol. Heart Circ. Physiol..

[38] Wang W.A., Groenendyk J., Michalak M. (2014). Endoplasmic reticulum stress associated responses in cancer. Biochim. Biophys. Acta.

[39] Krebs J., Agellon L.B., Michalak M. (2015). Ca^2+^ homeostasis and endoplasmic reticulum (ER) stress: An integrated view of calcium signaling. Biochem. Biophys. Res. Commun..

[40] McCullough K.D., Martindale J.L., Klotz L.O., Aw T.Y., Holbrook N.J. (2001). Gadd153 sensitizes cells to endoplasmic reticulum stress by down-regulating Bcl2 and perturbing the cellular redox state. Mol. Cell. Biol..

[41] Malhotra J.D., Kaufman R.J. (2007). The endoplasmic reticulum and the unfolded protein response. Semin. Cell Dev. Biol..

[42] Guo H.L., Hassan H.M., Ding P.P., Wang S.J., Chen X., Wang T., Sun L.X., Zhang L.Y., Jiang Z.Z. (2017). Pyrazinamide-induced hepatotoxicity is alleviated by 4-PBA via inhibition of the PERK-eIF2α-ATF4-CHOP pathway. Toxicology.

[43] Dudek J., Benedix J., Cappel S., Greiner M., Jalal C., Müller L., Zimmermann R. (2009). Functions and pathologies of BiP and its interaction partners. Cell. Mol. Life Sci..

[44] Liu X., Wang Z., Wang X., Yan X., He Q., Liu S., Ye M., Li X., Yuan Z., Wu J. (2021). Involvement of endoplasmic reticulum stress-activated PERK-eIF2α-ATF4 signaling pathway in T-2 toxin-induced apoptosis of porcine renal epithelial cells. Toxicol. Appl. Pharmacol..

[45] Puthalakath H., O’Reilly L.A., Gunn P., Lee L., Kelly P.N., Huntington N.D., Hughes P.D., Michalak E.M., McKimm-Breschkin J., Motoyama N. (2007). ER stress triggers apoptosis by activating BH3-only protein Bim. Cell.

[46] Yamaguchi H., Wang H.G. (2004). CHOP is involved in endoplasmic reticulum stress-induced apoptosis by enhancing DR5 expression in human carcinoma cells. J. Biol. Chem..

[47] Tao S.C., Yuan T., Rui B.Y., Zhu Z.Z., Guo S.C., Zhang C.Q. (2017). Exosomes derived from human platelet-rich plasma prevent apoptosis induced by glucocorticoid-associated endoplasmic reticulum stress in rat osteonecrosis of the femoral head via the Akt/Bad/Bcl-2 signal pathway. Theranostics.

[48] Nakagawa T., Zhu H., Morishima N., Li E., Xu J., Yankner B.A., Yuan J. (2000). Caspase-12 mediates endoplasmic-reticulum-specific apoptosis and cytotoxicity by amyloid-beta. Nature.

[49] Zhang Q., Liu J., Chen S., Liu J., Liu L., Liu G., Wang F., Jiang W., Zhang C., Wang S. (2016). Caspase-12 is involved in stretch-induced apoptosis mediated endoplasmic reticulum stress. Apoptosis.

[50] Singh M.P., Kang S.C. (2017). Endoplasmic reticulum stress-mediated autophagy activation attenuates fumonisin B1 induced hepatotoxicity In Vitro and In Vivo. Food Chem. Toxicol..

[51] Chen Q., Wang Y., Jiao F.Z., Shi C.X., Gong Z.J. (2019). Histone deacetylase 6 inhibitor ACY1215 offers a protective effect through the autophagy pathway in acute liver failure. Life Sci..

[52] Lin P., Chen F., Sun J., Zhou J., Wang X., Wang N., Li X., Zhang Z., Wang A., Jin Y. (2015). Mycotoxin zearalenone induces apoptosis in mouse Leydig cells via an endoplasmic reticulum stress-dependent signalling pathway. Reprod. Toxicol..

[53] Boussabbeh M., Ben Salem I., Prola A., Guilbert A., Bacha H., Abid-Essefi S., Lemaire C. (2015). Patulin induces apoptosis through ROS-mediated endoplasmic reticulum stress pathway. Toxicol. Sci..

[54] Yam G.H., Gaplovska-Kysela K., Zuber C., Roth J. (2007). Sodium 4-phenylbutyrate acts as a chemical chaperone on misfolded myocilin to rescue cells from endoplasmic reticulum stress and apoptosis. Investig. Ophthalmol. Vis. Sci..

